# Presence of a mutation in PSEN1 or PSEN2 gene is associated with an impaired brain endothelial cell phenotype in vitro

**DOI:** 10.1186/s12987-020-00235-y

**Published:** 2021-01-07

**Authors:** Snehal Raut, Ronak Patel, Abraham J. Al-Ahmad

**Affiliations:** Department of Pharmaceutical Sciences, Texas Tech University Health Sciences Center, Jerry H. Hodge School of Pharmacy, 1300 South Coulter Street, Amarillo, TX 79106 USA

**Keywords:** iPSCs, Blood–brain barrier, PSEN, Familial form of Alzheimer’s disease (FAD)

## Abstract

**Background:**

Alzheimer’s disease (AD) is the most common form of neurodegenerative disease. It is an irreversible condition marked by irreversible cognitive loss, commonly attributed to the loss of hippocampal neurons due to the formation of senile plaques and neurofibrillary tangles. Although the sporadic form is the most prevalent, the presence of familial form (involving several genes such as APP, PSEN1, and PSEN2) of the disease is commonly used as a model for understanding the pathophysiology of the disease. The aim of this study is to investigate the effect of a mutation on PSEN1 and PSEN2 genes on the BBB function using induced pluripotent stem cells (iPSCs).

**Methods:**

iPSC lines from patients suffering from a familial form of Alzheimer’s disease and harboring mutations in PSEN1 or PSEN2 were used in this study and compared to a control iPSC line. Cells were differentiated into brain microvascular endothelial cells (BMECs) following established differentiation protocols. Barrier function was assessed by measuring TEER and fluorescein permeability, drug transporter activity was assessed by uptake assay, glucose uptake and metabolism assessed by cell flux analyzer, mitochondrial potential by JC-1, and lysosomal acidification by acridine orange.

**Results:**

iPSC-derived BMECs from the FAD patient presenting a mutation in the PSEN1 gene showed impaired barrier function compared to the FAD patient harboring a mutation in PSEN2 and to the control group. Such impaired barrier function correlated with poor tight junction complexes and reduced drug efflux pump activity. In addition, both PSEN1 and PSEN2-BMECs displayed reduced bioenergetics, lysosomal acidification, autophagy, while showing an increase in radical oxygen species (ROS) production. Finally, PSEN1- and PSEN2-BMECs showed an elevated secretion of Aβ1–40 peptides compared to control-BMECs.

**Conclusions:**

Our study reports that iPSC-derived BMECs obtained from FAD patients showed impaired barrier properties and BMEC metabolism. In particular, mutation in the *PSEN1* gene was associated with a more detrimental phenotype than mutation in *PSEN2*, as noted by a reduced barrier function, reduced drug efflux pump activity, and diminished glucose metabolism. Therefore, assessing the contribution of genetic mutations associated with Alzheimer’s disease will allow us to better understand the contribution of the BBB in dementia, but also other neurodegenerative diseases.

## Background

Alzheimer’s disease (AD) is the most common form of dementia. It is a progressive and incurable neurodegenerative disease accounting and is the 6th cause of death in the United States. It is estimated that over 10% of the US senior population is diagnosed with AD [1].

The pathophysiology of the disease remains unclear. However, it is characterized by several features including the formation of amyloid plaques (rich in Aβ peptides) [2, 3] and hyperphosphorylation of Tau protein in the hippocampus region [4–7], resulting in neuronal cell death and ultimately propagating to the cortical regions surrounding the hippocampus. These two features provide sources for two hypotheses (the Aβ hypothesis and the Tau hypothesis, respectively) that have been used as potential targets for the development of therapies. Despite the important effort aimed to find a cure for such disease and the development of various animal models of the disease (including transgenic mice), translation from pre-clinical models into clinically relevant therapies remains the main pitfall in such effort [8, 9].

Recently, the use of induced pluripotent stem cells (iPSCs) as a tool to model Alzheimer’s disease (AD) has gained attraction [10–12]. Interestingly, such iPSCs are derived from patients suffering from the familial form of AD (FAD), characterized by mutations in genes associated with AD including amyloid precursor protein (APP) or presenilin (PSEN1, PSEN2). Although the pathogenesis of AD has been primarily focused on a neurocentric approach, recent studies have suggested the contribution of non-neuronal cells into the pathophysiology, including the blood–brain barrier (BBB).

Several studies by Zlokovic and colleagues have demonstrated a contribution of the BBB into the pathophysiology of AD [13–18]. In a recent review, Montagne and colleagues reported several in vivo studies performed in PSEN1 transgenic mice. Such animals displayed signs of an impaired barrier function in the form of microhemorrhage and increased permeability to tracers and large proteins (albumin, fibrin, IgG) [19]. Yet, the presence of similar features at the BBB of FAD patients harboring such mutations in the PSEN genes remains to be documented. The aim of this study is to document the effect of mutations in PSEN1 and PSEN2 genes on the barrier function using iPSCs derived from patients suffering from FAD [12, 20, 21]. Using the differentiation protocol initially developed by Shusta and colleagues [22, 23], this study investigated the effect of mutations on PSEN1 and PSEN2 genes on the barrier phenotype in iPSC-derived brain microvascular cells (BMECs).

## Materials and methods

### Cell culture and iPSC differentiation

Control (CS06iCTR), PSEN1 (CS40iFAD), and PSEN2 (CS08iFAD) iPSC lines used in this study were acquired from the Cedars-Sinai iPSC core (Los Angeles, CA). The PSEN1 iPSC line was isolated from a 56-year-old Caucasian male diagnosed with memory impairment and harbors an Ala246Glu mutation. The PSEN2 iPSC line was isolated from an 81-year-old Caucasian female diagnosed with progressive dementia and harbors an Asn141Ile mutation.

Undifferentiated iPSCs were maintained in hESC-grade Matrigel^®^ (Corning, Corning, NY) in presence of Essential 8 (E8) medium (Life Technologies, ThermoFisher, Waltham, MA) as previously described [24]. iPSC differentiation into BMECs occurred following the differentiation protocol previously published by our lab [24]. Briefly, cells were maintained in E8 for 5 days prior to differentiation, followed by 6 days in unconditioned medium [UM: Dulbecco’s modified Eagle’s medium/F12 with 15 mM HEPES (ThermoFisher), 20% knockout serum replacement (ThermoFisher), 1% non-essential amino acids (ThermoFisher), 0.5% Glutamax (ThermoFisher), and 0.1 mM β-mercaptoethanol (Sigma-Aldrich, St. Louis, MO, USA)] and 2 days in EC+/+ [EC medium (ThermoFisher) supplemented with 1% platelet-poor derived serum (PDS, Alfa-Aesar, ThermoFisher, Haverhill, MA, USA), 20 ng/mL human recombinant basic fibroblast growth factor (Tocris, Abingdon, UK), and 10 µM retinoic acid (Sigma-Aldrich)]. At day 8 of differentiation, cells were enzymatically dissociated (Accutase^®^, Corning) and seeded on tissue culture plastic surfaces (TCPS) coated with collagen (isolated from human placenta, Sigma-Aldrich)/fibronectin (bovine plasma, Sigma-Aldrich) at concentrations of 80 µg/cm^2^ and 20 µg/cm^2^ respectively. At day 9 of differentiation, iPSC-derived BMECs were maintained in EC−/− medium [EC medium supplemented with 1% PDS] for 24 h. Experiments were conducted at day 10 of differentiation.

### Immunofluorescence

Cells were quickly washed with ice-cold PBS and fixed in 4% paraformaldehyde (PFA, Electron Microscopy Sciences, Hatfield, PA, USA) and blocked for 30 min at room temperature (RT) in presence of PBS supplemented with 10% goat serum (ThermoFisher) supplemented with 0.2% Triton-X100 (Sigma). Cells were incubated overnight at 4 ºC in primary antibodies targeting BCRP (1:100, Millipore, RRID: AB_11213795), claudin-5 (1:100, Life Technologies, RRID: AB_2533200), GLUT1 (1:100, ThermoFisher, AB_10979643), GLUT3 (1:100, ThermoFisher, AB_2809974), GLUT4 (1:100, ThermoFisher, AB_11153908), MRP1 (1:100, Millipore, RRID: AB_2143819), occludin (1:100, Life Technologies, AB_2533101), P-gp (1:50, ThermoFisher, AB_1233253) and ZO1 (1:100, RRID:AB_2533938) diluted in 10% goat serum (PBSG). Primary antibodies detection occurred by incubation with goat-anti mouse Alexa Fluor^®^ 555-conjugated secondary antibody (Life Technologies) for 1 h at room temperature. Cells were observed at 200× magnification (20× long-distance dry objective) and acquired using a Leica DMi-8 inverted epifluorescence microscope (Leica Microsystems, Wetzlar, Germany). Background fluorescence was subtracted from unlabeled cells incubated with the secondary antibody only. Exposure time was set on the control iPSC-BMECs for each experiment and each protein of interest while maintained constant through the acquisition of other pictures in the PSEN1 and PSEN2 groups. Images were processed using ImageJ (Image J, NIH, Bethesda, MD). The average relative fluorescence was calculated by measuring the mean fluorescence from five microscopic fields (four cardinal directions and the middle of the well) and quantified using the built-in function in ImageJ. The average relative fluorescence was obtained for three independent biological replicates (three independent iPSC differentiation passages).

### TEER and permeability experiments

Barrier tightness was measured by assessing both transcellular electrical resistance (TEER) and fluorescein permeability (paracellular tracer). TEER was measured using an EVOHM STX2 chopstick electrode (World Precision Instruments, Sarasota, FL, USA). For each experiment, three measurements were performed for each insert, and the average resistance obtained was used to determine barrier function. Fluorescein permeability was assessed by incubating 10 µM sodium fluorescein (Sigma-Aldrich) in the donor (apical) chamber, with sampling in the donor (basolateral) chamber every 15 min for up to 60 m“in. Fluorescein permeability (P_e_) was calculated using the clearance slopes obtained by extrapolation using the following formula:

$${\frac{1}{\left({{{{\text{P}}}}_{e}}*{\text{S}}\right)}}= {\frac{1}{\left({{{{\text{P}}}}_{t}}*{\text{S}}\right)}}-{\frac{1}{\left({{{{\text{P}}}}_{f}}*{\text{S}}\right)}}{\text{ with {P}}_{e}}= {\frac{{\text{P}}_{e}*{{\text{S}}}}{{{\text{S}}}}}$$

P_t_ and P_f_ indicative of the clearance slopes of samples and blank (empty coated) filters, and S indicative of the insert surface area (cm^2^).

### Drug uptake assay

Cells were incubated in the presence of 10 µM Rhodamine 123 (P-gp substrate, Sigma), FL-BOPIDY (BCRP substrate, Sigma), or CM-DCFDA (MRP substrate, Sigma) for 1 h at 37 ºC followed by cell lysis using RIPA buffer (ThermoFisher). For assessing the contribution of efflux pump in the drug uptake, cells were pre-incubated for 1 h in presence of 5 µM cyclosporine A (CsA, P-gp inhibitor, Sigma), 1 µM Ko143 (BCRP inhibitor, Sigma), or 10 µM MK571 (MRPs inhibitor, Sigma) and maintained during the incubation with drug efflux substrate. Following incubation, cells were briefly washed with ice-cold PBS and lysed with RIPA buffer. Fluorescence in cell lysates was assessed using a SynergyMX^2^ ELISA plate reader (Bio-Tek, Winooski, VT, USA). Relative fluorescence units (RFU) were normalized against the total protein content and the protein levels were determined by bicinchoninic acid assay (BCA, ThermoFisher). Fluorescence values (expressed as relative fluorescence unit or RFU) obtained from cell lysates in the absence of inhibitor (named as controls) were normalized to the protein content and expressed as RFU/µg protein.

### Glucose uptake assay

Cells were incubated in the presence of [^14^C]-d-glucose (0.4 µCi/mL) for 1 h at 37 ºC.

Following incubation, cells were briefly washed with ice-cold PBS and lysed with RIPA buffer. In experiments involving GLUT1 inhibition, cells were pre-incubated in the presence of 10 µM glucose transporter inhibitor II (Millipore-Sigma, Danvers, MA) for 1-h prior to incubation with glucose. Radioactivity in cell lysates was assessed using a liquid scintillation cocktail (Scintisafe^®^ 30%, ThermoFisher) and quantified with a Beckman-Coulter LS6500 (Beckman-Coulter, Brea, CA). Glucose uptake levels were normalized by the total amount of protein in samples.

### Glycolytic flux and mitochondrial analysis

Glycolytic flux analysis was assessed using a Seahorse XFe-24 cell flux analyzer (Agilent Technologies, Santa Clara, CA). Cells were seeded at a density of 5 × 10^4^ cells/well on custom-designed 24-well plates (Agilent Technologies) at day 8 of differentiation and allowed to grow for 48 h. On the day of experiment, the EC−/− medium was replaced by a glucose-free medium provided with the glycolytic stress test kit (Agilent) for 2 h prior experiment. Cell medium was replaced once with glucose-free medium and initiated measurement of both the extracellular acidification rate (ECAR) and the oxygen consumption rate (OCR). At 20 min of incubation, 10 mM d-glucose was added in the incubation chamber, followed by the addition of 1 µM of oligomycin at 40 min and finally the addition of 100 mM 2-deoxy-d-glucose (2-DG) at 60 min timepoint, with measurements occurring until the 90th-min timepoint. The cell energetic profile was determined at the 3rd timepoint by plotting the ECAR and OCR values for each well and delimited using the quadrants established by the manufacturer. The assessment of the glycolytic parameters was performed by the Seahorse Wave software data analyzer (Agilent), using the ECAR values reported during the experiments. Briefly, the glycolysis parameter was obtained by measuring changes in the ECAR following the addition of d-glucose, whereas the glycolytic reserve was determined by measuring the ECAR values following the oligomycin treatment. The glycolytic capacity was determined by subtraction of the glycolytic reserve minus the glycolysis. Finally, the non-glycolytic acidity was determined from the ECAR values following the 2-DG treatment.

In mitochondrial stress assays, changes in oxygen consumption rates were accomplished using the Mito Stress Test kit assay. Cells were maintained in assay medium (DMEM) for 1 h at 37 ºC (0% CO_2_) prior to the performance of the assay as recommended by the manufacturer. The assay was initiated by measuring changes in the OCR values for 20 min to determine the basal respiration, followed by treatment with 1.5 µM oligomycin (complex V ATP synthase inhibitor). This step allows us to the ATP-linked respiration (by measuring the difference in OCR before and after oligomycin treatment). Following treatment with oligomycin, cells are treated with 0.5 µM FCCP (uncoupling agent) that allows the measurement of the spare capacity (OCR after FCCP treatment—basal respiration). Finally, 0.5 µM rotenone/antimycin A (R/AA, complex I and III) allows the measurement of the maximal respiration (difference in OCR before and after R/AA treatment), calculation of the non-mitochondrial oxygen consumption (OCR measurement at the last timepoint), and proton leak (OCR following oligomycin treatment—OCR at last timepoint).

### JC-1 flow cytometry and live imaging

At day 10 of differentiation, cells were enzymatically dissociated with Accutase^®^. Cells were resuspended and centrifuged and resuspended in medium containing 5 µM JC-1 dye (ThermoFisher) for 30 min at 37 ºC. In experiments involving FCCP treatment, cells were simultaneously treated with 50 nM FCCP. Following incubation with JC-1 dye, cells were washed by centrifugation and resuspension in 200 µL PBS for flow cytometry analysis. In experiments involving live imaging, cells were incubated in presence of 100 µL of JC-1 reagent (Cayman Chemicals, Ann Arbor, MI) and incubated for 20 min at 37 ºC/5% CO_2_.

Under physiological conditions, the high mitochondrial membrane potential (MMP) results in the formation of “J-aggregates”, which accumulates within the mitochondria, while emitting a fluorescence around 590 nm (red). Under mitochondrial stress condition, JC-1 remains as monomers and emit a fluorescence around 530 nm (green). Live cells were observed under the Leica DMi-8 microscope, with the use of the 485/535 nm for impaired cells and 540/570 nm for healthy cells to detect JC-1 aggregates, whereas cell events in the flow cytometry were counted at PMTs capturing fluorescence signals in the fluorescein isothiocyanate (FITC) and phycoerythrin (PE) emission ranges respectively. The fluorometric ratio of JC-1 was used as an indicator of mitochondrial dysfunction by assessing changes in mitochondrial membrane potential. In experiments involving FCCP treatment (an uncoupling agent disrupting the mitochondrial oxidative phosphorylation), cells were simultaneously treated with 50 nM FCCP for 1-h prior to incubation with JC-1 dye.

### Acridine orange (AO) flow cytometry

At day 10 of differentiation, cells were maintained for 24 h in EC^−/−^ medium or in serum-free EC medium to induce serum starvation. Following such treatment, cells were enzymatically dissociated with Accutase^®^. Cells were resuspended and centrifuged and resuspended in medium containing 1 µg/mL acridine orange (AO, Sigma-Aldrich) dissolved in PBS and allowed to stain for 15 min, following the protocol of Thome and colleagues [25]. Fluorescence detection in samples was performed using a FACSVerse^®^ flow cytometer (BD Biosciences, San Jose, CA). Fluorescence PMTs were calibrated on unstained cells and set for the remaining of the experiments. Quadrants were set on control cells, with events expected to occur as FITC^high^ and PE^high^.

Under physiological condition, AO accumulates inside acidic vesicular organelles (AVOs, such as autophagic lysosomes) and emit a green fluorescence under such conditions. However, in late-stage autophagy, AO can dimerize and shift its emission range into a red fluorescence. Therefore, the quantification of both AO monomer fluorescence in the green (FITC) and red (PE) channels is indicative of functional autophagy and the presence of AVOs. Induction of autophagy by serum starvation will result in an increase in such dimerization process and ultimately a shift in the emission pattern with a decrease in green fluorescence and an increase in red fluorescence.

### Lysosensor(R) live imaging

Live cells were incubated in presence of 1 µM Lysosensor-Green DND 189 for 5 min, followed by a brief wash with ice-cold PBS and fixation with 4% paraformaldehyde. Cells were counterstained with 300 nM DAPI solution and immediately processed for imaging under the Leica DMi-8 inverted fluorescence microscope at 20×.

### Radical oxygen species assays

At day 10 of differentiation, intracellular radical oxygen species (ROS) was assessed using both a qualitative and quantitative approach. The qualitative approach in assessing ROS production was performed using CellROX(R) assay (ThermoFisher Scientific). CellROX(R) reagent was added to live cells at a final concentration of 5 µM to the medium and incubated for 30 min at 37 °C/5% CO_2_. The medium was removed, and cells were quickly washed with PBS. Cells were observed at 20× magnification, and images were acquired using a Leica DMi-8 inverted fluorescence microscope (Leica Microsystems). The quantitative approach in assessing ROS production was performed using a DCFDA-based cell kit (Cayman Chemicals), following the instruction provided by the vendor. DCFDA fluorescence was measured using SynergyMX2 ELISA plate reader (Bio-Tek). DCFDA fluorescence intensity in CTR90F BMECs was used as a baseline value for comparison to the PSEN BMECs.

### Cell viability

To study the effect of such mutations on cell viability, a LIVE/DEAD™ Viability/Cytotoxicity Kit (Invitrogen) was used. A calcein-AM/ethidium homodimer-1 (EthD-1) assay was performed based on the manufacturer’s protocol. Briefly, cells were stained by a dye solution consisted of 0.5 µL of Calcein AM and 0.5 µL of ethidium homodimer-1 per mL PBS. Cells were observed after 20 min of incubation at 20× magnification, and fluorescence was acquired using a Leica DMi-8 inverted fluorescence microscope (Leica Microsystems, Wetzlar, Germany). Ethidium homodimer-1 enters into cells with compromised cell membranes and subsequently intercalates with nucleic acids. Thus, dead cells have red fluorescence. Calcein-AM penetrates living cell membranes and produces green fluorescence by cleavage of cytoplasmic esterase.

### Aβ1–40 and Aβ1–42 ELISA

Cell conditioned medium from BMECs monolayers grown at Day 10 were collected and immediately frozen at − 80 ºC. Aβ1–40 and 1–42 levels in supernatants were measured using their respective ELISA Quantikine(R) kits (R&D Systems, Minneapolis, MN).

In experiments involving cell homogenates, cells were briefly washed with PBS followed by homogenization using 200 µL RIPA buffer (ThermoFisher) supplemented with 1× protease inhibitors cocktail (ThermoFisher). Cells were maintained on ice for 10 min, followed by centrifugation at 14,000 rpms for 10 min at 4 ºC. The supernatant of such lysates was collected and immediately frozen at − 80 ºC pending analysis. Cell lysates protein concentrations were measured using BCA assay and normalized (using the diluent provided by the kit) to achieve a final protein concentration of 1 µg total protein/µL if necessary. 200 µL of such normalized samples were loaded onto the ELISA plate.

### Statistics

Data are represented as mean ± S.D. from at least three independent experiments. Statistical analysis was performed using one-way or two-way analysis of the variance (ANOVA) using parametric tests. Group comparisons were performed against the control iPSC group, both in one-way and two-way ANOVA posthoc analysis. Statistical analysis was performed using GraphPad Prism 8.0 (GraphPad Software, La Jolla, CA). A P-value lesser than 0.05 (*P < 0.05*) was considered as indicative of a statistical difference between one or more groups.

## Results

### BMECs from PSEN1 patient showed impaired barrier function

**Fig. 1:**
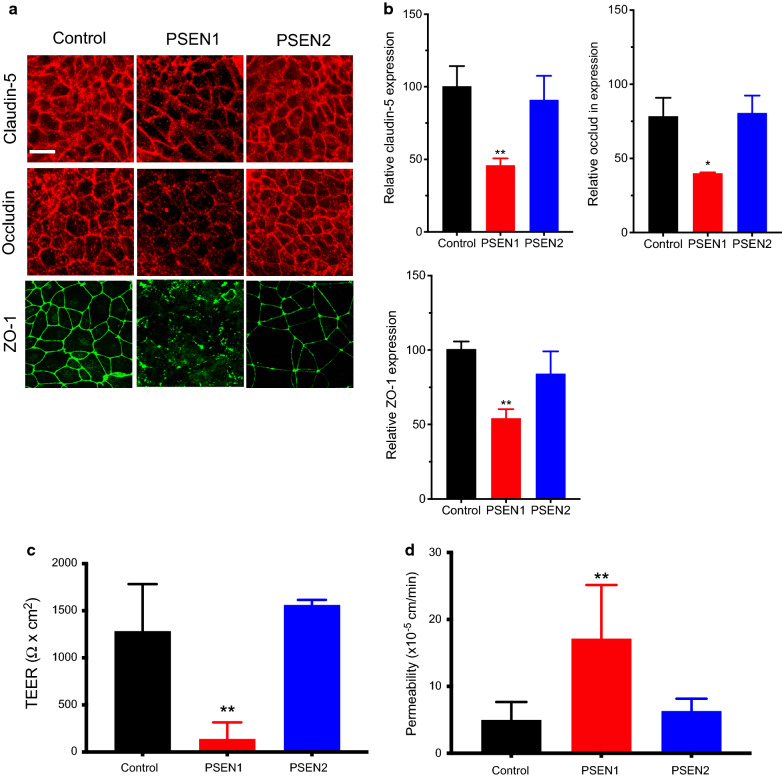
PSEN1-BMECs show impaired tight junctions and barrier function. **a** Representative micrograph picture of claudin-5, occludin, and ZO-1 immunofluorescence in iPSC-derived BMECs differentiated from control, PSEN1, and PSEN2 patients. Scale bar = 50 µm. **b** Semi-quantitative analysis of claudin-5 and occludin protein expression. Protein expression was quantified using fluorescence intensity obtained in random fields. Note the relative lower expression of claudin-5 and occludin in the PSEN1 group compared to PSEN2 group. **c** TEER and **d** fluorescein permeability values in iPSC-derived BMECs at day 10 of differentiation. Note the poor TEER value and higher permeability in the PSEN1 group compared to other groups. N = 3/group, ** Denotes P < 0.01 compared to controls

The first goal of this study aimed to assess the presence of a BMEC phenotype in iPSC derived BMECs from FAD patients compared to control iPSCs (Fig. 1). Thus, the expression of tight junction (TJ) proteins with BMECs was assessed using immunocytochemistry (Fig. 1a). Interestingly, PSEN1-BMECs displayed a lower immunoreactivity to claudin-5, occludin and ZO-1 (Fig. 1a) accompanied by a decreased immunostaining at the cell borders. A semi-quantitative analysis of relative protein expression (by measuring the average fluorescence intensity) (Fig. 1b) suggested an overall decreased protein expression in PSEN1-BMECs, whereas PSEN2-BMECs showed no major differences to the control iPSC line. The expression pattern of these TJ proteins in control-BMECs was comparable to other iPSC lines obtained from control (healthy) patients [24].

To better correlate the deficiency in the expression of TJ complexes with an impaired barrier function, changes in TEER and fluorescein permeability were measured in all three cell lines (Fig. 1c, d). PSEN1-BMECs showed impaired barrier function compared to the two other iPSC lines, as a significantly lower TEER (~ 150 Ω cm^2^) and higher fluorescein permeability. On the other hand, TEER and permeability values reported in the control and PSEN2-BMECs were comparable to control iPSC lines previously used by our group [24], as these cells displayed tight monolayers (> 1000 Ω cm^2^) and low paracellular permeability to fluorescein (10^− 5^cm/min). Such impaired phenotype appeared not limited to BMECs, as iPSC-derived neurons originated from PSEN1 iPSCs displayed an impaired formation of maturing neurons as represented by the formation of neurites compared to control and PSEN2-neurons (Additional file 1: Fig. S1). Taken together our data suggests that PSEN1, but to a lesser extent, PSEN2, may impair the barrier function in BMECs.

### PSEN1-BMECs have impaired drug efflux pumps activity

**Fig. 2:**
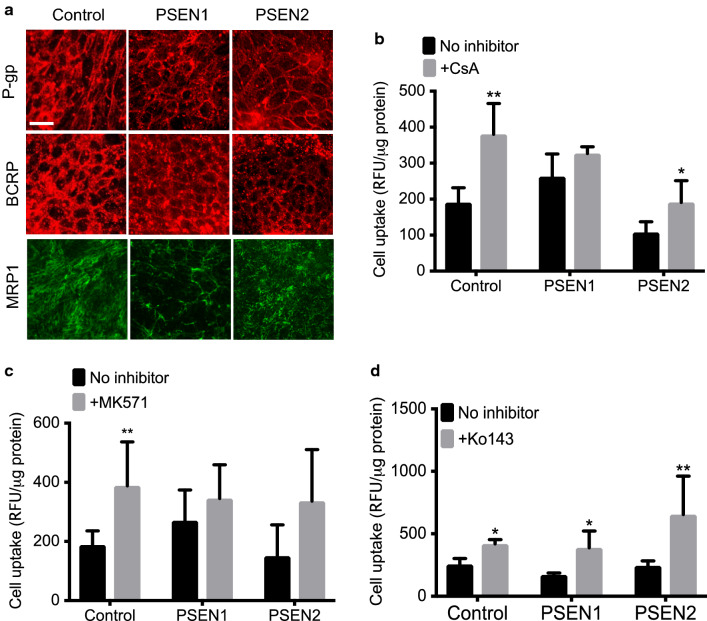
PSEN1-BMECs show lower P-gp and MRP1 activity than control-BMECs and PSEN2-BMECs. **a** Representative micrograph pictures of P-gp, BCRP and MRP1 immunostaining in BMECs derived from the control, PSEN1, and PSEN2 iPSC lines. Scale bar = 50 µm. Cell uptake assay of drug efflux substrate for assessing P-gp (**b**), BCRP (**c**) and MRPs (**d**) activity. Note the higher P-gp and MRPs efflux substrate uptake in PSEN1-BMECs compared to the two other iPSC lines, as well as an absence of increased drug efflux substrate in PSEN1 following P-gp and MRPs inhibition by cyclosporine A and MK571 respectively. N = 3/group, * and ** denote P < 0.05 and P < 0.01 respectively

To further investigate the BMEC phenotype associated with FAD, changes in ABC transporters expression (by immunocytochemistry) and activity (by drug uptake assay) were documented in this study (Fig. 2). All these three iPSC-derived BMECs displayed the expression of common ABC transporters expressed at the BBB (Fig. 2a), including ABCB1 (P-gp), ABCC1 (MRP1), and ABCG2 (BCRP). No significant differences were observed in the relative expression of these transporters, albeit ABCB1 and ABCC1 displayed a slightly lower immunoreactivity in PSEN1-BMECs. To further investigate the possible differences in drug efflux pump activity between the different iPSC lines used, fluorescence substrate uptake assays were performed in all three cell lines. PSEN1-BMECs showed higher drug uptake levels for rhodamine-123 (Fig. 2b, a P-gp substrate) and DCFDA (Fig. 2c, an MRPs substrate), and confirmed by the relative absence of change in cellular uptake following treatment with cyclosporine A (CsA, a P-gp inhibitor) or MK571 (a pan-MRP inhibitor). In contrast, no significant differences were observed in regard to BCRP activity. In conclusion, the mutation in the *PSEN1* gene may impair the activity of certain drug efflux transporters.

### PSEN1 and PSEN2-BMECs display impaired glucose uptake and metabolism

**Fig. 3:**
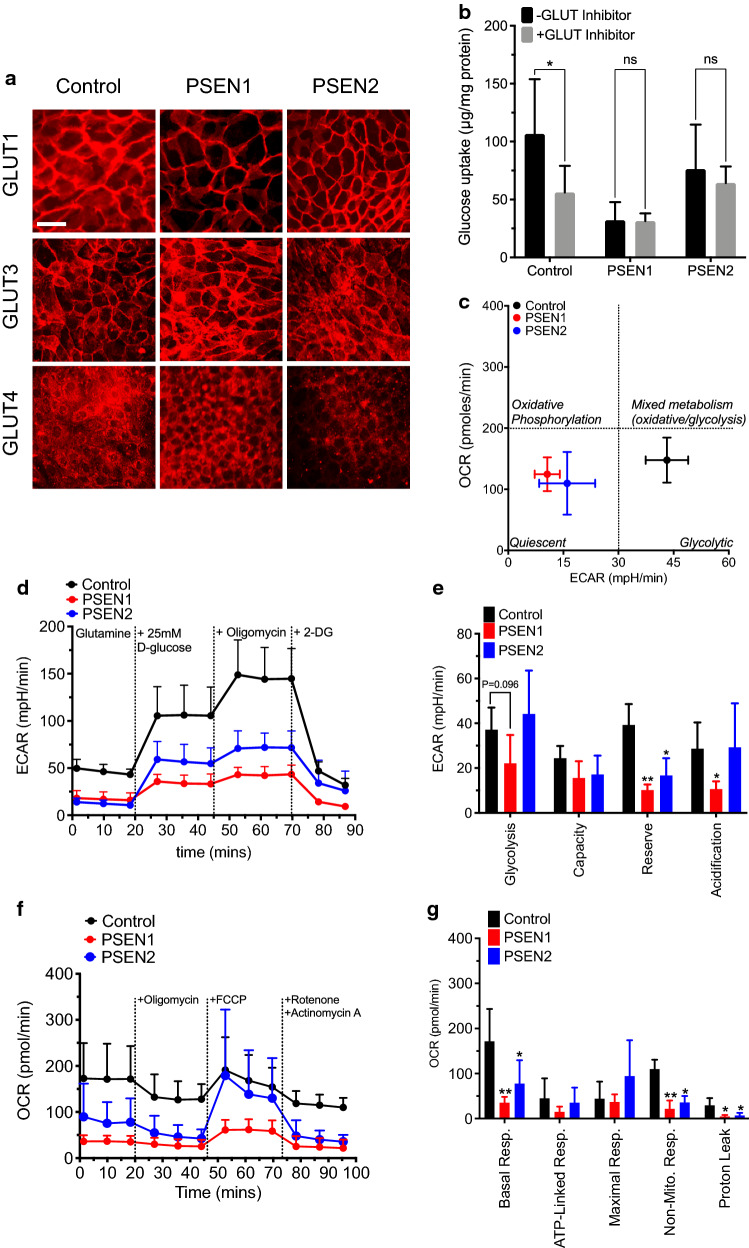
PSEN1 mutation impacts glucose uptake and bioenergetic profile in BMECs monolayers. **a** Representative micrograph pictures of GLUT1, GLUT3 and GLUT4 immunostaining in BMECs derived from the control, PSEN1, and PSEN2 iPSC lines. Scale bar = 50 µm. **b** Glucose uptake assay in iPSC-BMECs in absence or presence of 10 µM Glucose Transporter Inhibitor II. Note the absence of glucose uptake inhibition in both the PSEN1 and PSEN2 iPSC lines. **c** Glycolytic flux analysis. Representative ECAR diagram following treatment with various inhibitors. Cells were incubated for 2 h in a glucose-free medium prior to the onset of the experiment. Cells were maintained in medium with l-glutamine, and subsequently given 10 mM d-glucose, followed by incubation with 1 µM oligomycin (mitochondria respiratory chain inhibitor) and 2-deoxyglucose (100 mM). **d** Energy consumption profile of iPSC-BMECs. OCR denotes oxygen consumption rate, ECAR denotes extracellular acidification rate. Note the shift of metabolic activity from “glycolytic” to “quiescent” phenotype. **e** Glycolytic parameters extrapolated. Note the lower glycolytic capacity and reserve in PSEN1 and PSEN2 iPSC-BMECs compared to control iPSC-BMECs, whereas PSEN1 showed lower glycolysis and non-glycolytic acidification rate. **f** Oxygen consumption profile in iPSC-BMECs following mitochondrial stress challenge. Cells were challenged with 1.5 µM oligomycin (ATP synthase inhibitor), followed by treatment with 0.5 µM FCCP (uncoupling agent) and 0.5 µM rotenone/antimycin A (inhibitor of the complex I and III of the mitochondrial respiratory chain). Note the basal respiration rate (measured prior oligomycin treatment) observed in both PSEN1- and PSEN2-BMECs, as well as the limited effect of oligomycin on ATP production in PSEN1-BMECs. N = 3/group, * denotes P < 0.05 versus control group, # denotes P < 0.05 versus non-inhibited group

Next, changes in glucose uptake and metabolism between iPSC lines were assessed (Fig. 3). No significant changes in glucose transporter isoforms (GLUT1, GLUT3, GLUT4) at the BBB were observed (Fig. 3a). However, PSEN1-derived BMECs showed a lower glucose uptake (Fig. 3b) compared to controls and PSEN2-BMECs. PSEN2-BMECs showed a lower but non-significant decrease in glucose uptake compared to the control group. In addition, both PSEN1-BMECs and PSEN2-BMECs failed to show inhibition of glucose uptake following treatment with glucose transporter inhibitor II (GTI). Notably, a similar pattern was observed with iPSC-astrocytes (Additional file 1: Fig. S2), as PSEN1-astrocytes showed lower glucose uptake than control-astrocytes. However, all three groups showed significant decreases in glucose uptake following treatment with GTI. To investigate the impact of such impaired glucose uptake on cell metabolism, we investigated changes in glycolysis in iPSC derived BMECs using a cell flux analyzer (Fig. 3c–e). All three cells were characterized as “glycolytic” cells due to their relatively low oxygen consumption rate (OCR) compared to the extracellular acidification rate (ECAR). However, both PSEN1-BMECs and PSEN2-BMECs showed a lower basal extracellular acidification rate (ECAR) compared to control-BMECs. Both PSEN-BMECs showed a metabolic phenotype considered “quiescent”, compared to a “glycolytic” phenotype observed with control-BMECs. A detailed analysis of the glycolytic stress assay (Fig. 3d, e) showed a decrease in glycolysis and non-glycolytic activity only in PSEN1-BMECs, whereas a decreased glycolytic capacity and glycolytic reserve were observed in both PSEN-BMECs.

To further identify if this energetic failure was exclusive to the glycolytic pathway, changes in mitochondrial activity was assessed by challenging the mitochondrial function using the “mitochondrial stress kit” (Fig. 3f, g). Both PSEN1 and PSEN2-BMECs were displaying a lower respiration rate (basal OCR) than control-BMECs, as well as non-mitochondrial respiration, indicative of an impaired mitochondrial function. Interestingly, PSEN2-BMECs showed a higher maximal respiratory mitochondrial function compared to the two other cell lines.

In conclusion, mutations in PSEN1 or PSEN2 genes may impair energy production in BMECs by affecting both glycolysis and mitochondrial respiration.

### PSEN1 mutation impairs mitochondrial membrane potential and lysosomal acidification

**Fig. 4:**
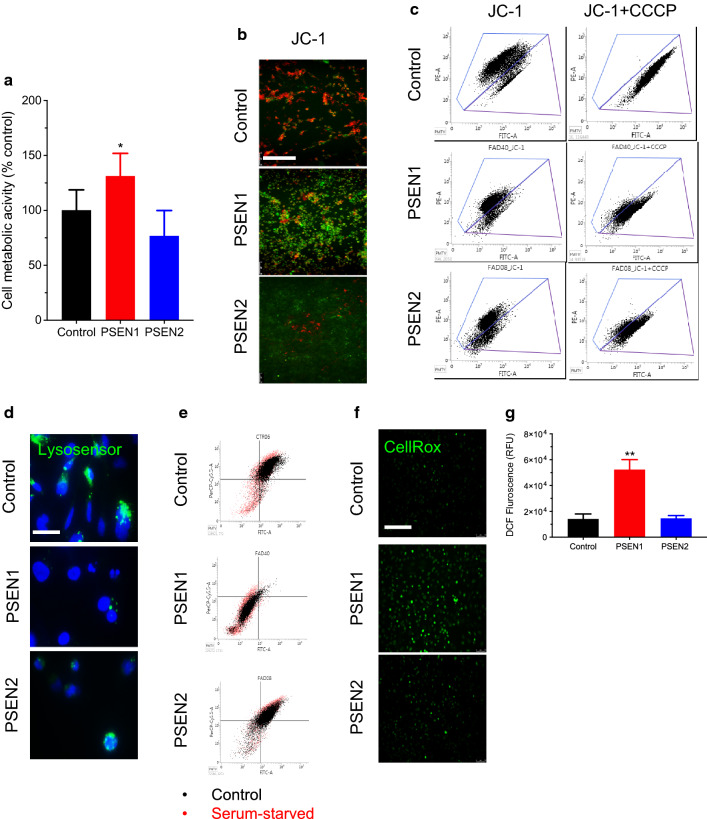
PSEN1 abnormally high cell metabolic activity is coupled with impaired mitochondrial function. **a** MTS assay in iPSC derived BMECs. Cells were incubated in presence of MTS for 2 h. The MTS-conditioned medium was recovered and measured by spectrophotometry. Note the higher cell metabolic activity observed in PSEN1-BMECs compared to control-BMECs and PSEN2-BMECs. **b** JC-1 live imaging micrograph picture. Note the difference in JC-1 aggregation (as seen by the change in the emission fluorescence wavelength) with control cells showing predominantly JC-1 aggregates (red). In contrast, PSEN1-BMECs showed mostly JC-1 monomers (green) indicative of impaired mitochondrial membrane potential. Scale bar = 200 µm. **c** JC-1 flow cytometry analysis. The physiological mitochondrial membrane potential (MMP) is reflected as a “high-red”/low-green” JC-1 dye fluorescence. Inhibition of such membrane potential using an uncoupling agent (CCCP) results in the disruption of the proton gradient, resulting in the loss of the “red” fluorescence and a shift of the JC-1 fluorescence towards the “green” fluorescence. Note the absence of population shift occurring in PSEN1 (and to a lesser extent in PSEN2) BMECs following treatment with CCCP compared to the untreated cell group, suggesting an impaired mitochondrial membrane potential impairment. **d** Representative micrograph pictures of Lysosensor-Green(R) in iPSC-derived BMECs, counterstained with DAPI (nuclei stain, blue). Note the decreased Lysosensor-positive nuclei, indicative of impaired lysosomal acidification. Scale bar = 10 µm. **e** Flow cytometry profile of iPSC-BMECs following autophagy induction (by serum-starvation) for 24 h, followed by incubation with acridine orange. Note the cell events distribution in the control group (high FITC/high PerCP) indicative of acidic lysosomes. Such distribution is drastically changed in PSEN1-BMECs. **f** Representative CellRox(R) staining in iPSC-BMECs monolayers using live imaging. Note the significant increase in CellRox(R) fluorescence in PSEN1-BMECs indicative of an elevated ROS production in such cells. Scale bar = 200 µm. **f** Quantitative analysis of ROS levels using DCFDA fluorometric assay. Note the exacerbated ROS levels in PSEN1-BMECs compared to the two other cell types. N = 3/group, * and ** denote P < 0.05 and P < 0.01 versus the control group

PSENs are commonly associated with their function within the γ-secretase complex. However, recent studies highlighted the contribution of PSENs to other biological functions, including autophagy and mitophagy [26–28]. In particular, mutations in PSENs have been documented to be associated with impaired mitochondrial Ca^2+^ homeostasis and impaired mitochondrial function [29, 30]. Therefore, we investigated changes in the mitochondrial function and autophagy in iPSC-derived BMECs (Fig. 4) to report similar outcomes than reported in the literature in non-endothelial cells. Firstly, changes in cell metabolic activity were assessed in iPSC-BMECs monolayers using an MTS assay (Fig. 4a). Interestingly, PSEN1-BMECs showed a higher cell metabolic activity than control and PSEN2-BMECs. Similar outcomes in terms of MTS levels were observed in iPSC-derived neurons (Additional file 1: Fig. S2B), whereas no differences in iPSC-derived astrocytes were reported. To confirm if such differences in cell metabolic activity were due to changes in mitochondrial potential, changes in JC-1 fluorescence profile were performed, using immunofluorescence and flow cytometry (Fig. 4b, c). Control BMECs showed healthy mitochondria, as JC-1 aggregates (red) were observed under fluorescence. Such fluorescence pattern is considered indicative of a normal mitochondrial membrane potential (MMP). However, PSEN1-BMECs displayed mostly JC-1 monomers (green), indicative of impaired MMP. PSEN2 on the other hand showed a profile in between control and PSEN1 BMECs.

To provide a quantitative analysis of such observations, flow cytometry experiments for JC-1 were performed (Fig. 4c). Under resting conditions, control BMECs showed most cell events as high “red” (PE emission filter) fluorescence and low “green” (FITC emission filter), suggesting the presence of mitochondrial membrane potential. Following treatment with CCCP (a mitochondria uncoupler), JC-1 fluorescence emission shifted from “red” to “green” wavelength, with the majority of cell events reported in the control iPSC line occurred in the “green” emission filter. In contrast, PSEN1-BMECs and PSEN2-BMECs showed an overall lower “red” fluorescence and higher “green” intensity compared to control, suggesting a possible impairment in the MMP. Treatment with CCCP failed to fully shift the cell events towards the “green” filter, further evocating a possible impairment in the mitochondria cell membrane potential.

As mutations in PSEN1 correlated with impaired mitophagy in vitro and in vivo in non-BMECs cells, we investigated the possible impairment of cell autophagy in FAD cells using Lysosensor^®^ Green and Acridine Orange (AO), two fluorescent dyes monitoring changes in lysosomal pH (Fig. 4d, e respectively). Control BMECs displayed the presence of dense green peri-nuclear punctate indicative of acidified lysosomes. In contrast, the lack of Lysosensor^®^ Green punctate in PSEN1-BMECs was indicative of weak lysosomal acidification. PSEN2-BMECs appeared similar to control BMECs.

To confirm the impaired lysosomal acidification observed in PSEN1-BMECs following treatment with Lysosensor^®^ Green, cells were treated with AO and quantified using flow cytometry (Fig. 4d). Control BMECs were characterized by the presence of predominant high “red” (emission in PerCP filter) /high “green” (emission in FITC filter) cellular events. Such a feature is commonly accepted as indicative of acidic lysosomes [25]. In contrast, PSEN1-BMECs showed a notable decrease in acidic lysosomes compared to control-BMECs, as the majority of such events are recorded as “low red/low green” events. PSEN2-BMECs showed an intermediate pattern, as a tail in the “low red/low green” quadrant was observed. Induction of autophagy by serum starvation triggered a shift from “green” to “red” fluorescence, suggestive of an increase in acidic vesicular organelles (AVOs), as reported by Thome and colleagues [25]. Such an increase was reported in all three groups. In summary, FAD-associated mutations may impair mitochondrial membrane potentials and autophagy in BMECs.

### Mutation in PSEN1 is associated with increased ROS production

Finally, differences in free radical oxygen species (ROS) levels were assessed in iPSC-derived BMECs using CellRox(R) and DCFDA (Fig. 4f, g). PSEN1-BMECs showed a relatively high oxidative stress status, as compared to control and PSEN2-BMECs (Fig. 4f) indicative of higher ROS basal production in live cells. Using DCFDA as a quantitative fluorescent dye for ROS production (Fig. 4g), PSEN1-BMECs showed a significantly higher DCF level compared to control BMECs, as DCF levels were about 3-times higher than control. In contrast, PSEN2-BMECs showed similar levels to control. Results were further validated as treatment of control BMECs with 1 mM pyocyanin (Additional file 1: Fig. S2C), a known toxin capable to induce oxidative stress in human cells [31], resulted in a fourfold increase in DCF fluorescence compared to untreated cells. On the other hand, treatment with 300 mM *N*-acetylcysteine (NAC) showed no differences versus untreated groups. Taken together, such mutation in the PSEN1 gene may be associated with a higher oxidative stress status in BMECs.

### PSEN1 and PSEN2 significantly increase amyloidogenesis in BMECs

**Fig. 5:**
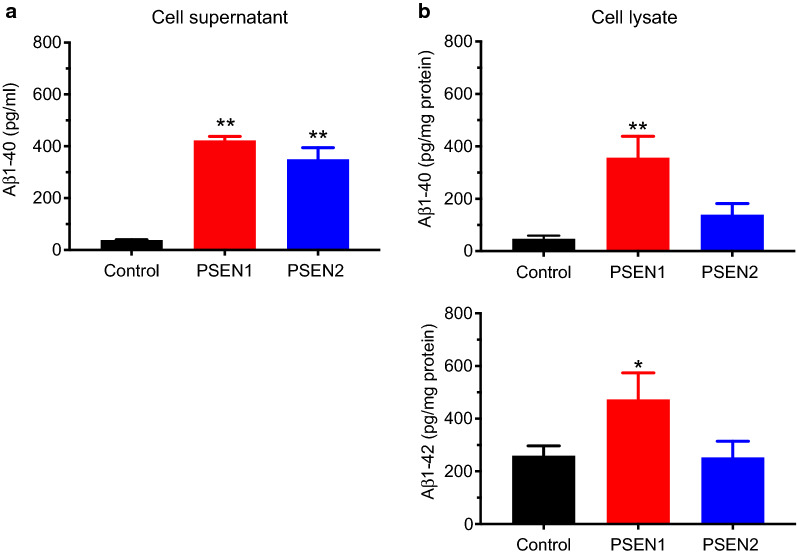
PSEN mutants display an increased Aβ1–40 secretion and intracellular concentrations. **a** Aβ1–40 secretion levels in the cell conditioned medium. Cells were allowed to condition in such medium for 24 h before harvesting. Note the extremely high levels in both PSEN1-BMECs and PSEN2-BMECs compared to control. Secreted Aβ1–42 was below detection limits for all three cell lines (data not shown). **b** Aβ1–40 and Aβ1–42 in cell homogenates. Note the presence of both Aβ1–40 and 1–42 in PSEN1-BMECs, at an amount significantly higher than control. N = 3/group, * and ** denote P < 0.05 and P < 0.01 versus the control group. # and ## denote P < 0.05 and P < 0.01 between PSEN1 and PSEN2 groups

To conclude this study, levels of both Aβ1–40 and Aβ1–42 were assessed in cell-conditioned medium and homogenates (Fig. 5). Levels of Aβ1–40 in control BMEC supernatants (Fig. 5a) were around 40 pg/mL, in the same range as secretion levels observed in cultured peripheral blood leukocytes and human motor cortex (values provided by the kit manufacturer). However, both PSEN1 and PSEN2-BMECs displayed a tenfold higher level than control BMECs, with values of 400 and 350 pg/mL respectively. No detectable levels of Aβ1–42 were measured in the cell-conditioned medium of any BMECs monolayers (data not shown).

In addition to secreted Aβ peptides, the presence of intracellular Aβ was examined in BMECs cell homogenates (Fig. 5b). Aβ1–40, and to a lesser extent Aβ1–42, was at a higher concentration in PSEN1-BMECs compared to control. Although PSEN2-BMECs showed a higher level of intracellular of Aβ1–40 compared to control BMECs, no differences in Aβ1–42 was noted. In conclusion, mutation in PSENs results in higher amyloidogenesis compared to control BMECs, mostly resulting in the generation of Aβ1–40 peptides.

## Discussion

Alzheimer’s disease (AD) is the most common form of dementia and represents the 6th leading cause of death in the United States. Because aging is the first risk factor by its preponderance, increased occurrence amongst an aging population raises important public health issues. The etiology of AD is commonly divided into the sporadic form of the disease (which accounts for > 90% of total cases), and the familial form of Alzheimer’s disease (FAD, which accounts for 5–10% cases) [32]. Several genes have been associated with FAD (e.g., *APP*, *PSEN1*, *PSEN2*…); however, mutations in the PSEN1 gene (with over 300 mutations) are considered as the most common mutations in FAD patients according to Alzforum (https://www.alzforum.org/mutations/psen-1) and the existing literature [6, 33–36].

In a recent review by Montagne and colleagues [19], several studies reported elements indicative of a compromised BBB function both in pre-clinical models (PSEN1 transgenic mice) and in patients. Yet, evidence of a direct association between mutations in PSEN1 and impairment of the BBB function remains limited.

In this study, using iPSCs derived from patients suffering from FAD, we investigated the possible link between PSENs and impaired barrier function. This study suggests that mutations in the PSEN1 gene may be detrimental to barrier function, as we reported a worsened barrier tightness in BMECs, decreased glucose uptake and metabolism, and impaired mitochondrial membrane potential. Such findings seem to correlate with a recent study by Searson and colleagues using two iPSC lines clones (iPSCAD6 and iPSCAD10) originating from the same patient (Coriell ID #AG06848, adult fibroblast from a 56-years old female) sharing the same mutation in PSEN1 (Ala246Glu) [35]. Our study is in agreement with that study, as we both observed that PSEN1-BMECs BMECs showed poor barrier function (as measured by TEER and permeability with a paracellular marker) compared to control BMECs. Furthermore, our group showed that MRP-mediated efflux in PSEN1-BMECs was also affected, as was glucose metabolism (glucose uptake, glycolysis), as well as glucose metabolism, mitochondrial function, and lysosomal acidification. Although our study highlighted such dramatic changes in PSEN1, we cannot exclude that similar outcomes may occur in PSEN2 BMECs as well, albeit at a much slower rate. Therefore, further investigations comparing PSEN1 and PSEN2 mutations and their effects on the BBB (using both in vitro and in vivo models) could provide some insights on the differential effects of PSEN1 and PSEN2 mutations at the BBB.

A major limitation of this study is highlighted by the limited number of FAD iPSC lines available from patients in public repositories. Because of such limitations, we cannot completely exclude that the impairment observed in the PSEN1 iPSC line may be inherent to the iPSC clone used in this study, although our findings aligned with the existing literature [35]. Hence, our future goal is to confirm these initial findings by the inclusion of additional iPSC lines from patients harboring mutations in both PSEN1 and PSEN2 lines.

A particular feature observed in our study was the lower glucose uptake in both PSEN1 and PSEN2 iPSC lines compared to control. Such lower uptake was accompanied by a lack of response to GLUT inhibition by GTI, and by a much lower ECAR value and glycolytic capacity compared to controls. Although the overall expression of GLUT1 appeared unchanged, we cannot exclude a possible impaired GLUT1 activity due to intrinsic factors. GLUT1 has been documented to have a particular interaction with Aβ, as a recent study by Zlokovic and colleagues reported a worsened outcome in AD transgenic mice crossed with Slc2a1^+/−^ deficient mice [15]. We cannot exclude that the decrease in glucose uptake (and other transporters activity) may be the result of mistrafficking, as recently reported by Selbach and colleagues [37]. Hence, our future direction will be to further investigate the relationships and interactions between Aβ peptides and GLUT1. We cannot exclude that compensatory mechanisms may occur by the recruitment of other glucose transporter isoforms. Our group and others previously documented the presence of GLUT3 and GLUT4 at the blood–brain barrier [38–41]. Notably, preliminary data obtained by our group suggest a higher expression of *SLC2A3* and *SLC2A4* genes at mRNA levels in PSEN1-BMECs compared to control, whereas PSEN2-BMECs shows a higher gene expression of *SLC2A4* compared to control (*data not shown*). Such preliminary data elicit the need for better documentation of glucose metabolism in brain endothelial cells.

The effect of PSEN1 and PSEN2 on BBB maturation and maintenance is intriguing. Both proteins are known to be part of the γ-secretase complex, which ultimately drives the formation of Aβ peptides. ELISA data obtained in our model showed that both PSEN1 and PSEN2 mutants displayed a much higher Aβ1–40 secretion than control BMECs, indicative of increased amyloidogenesis in these mutants. Such secretion profile seems to be mostly biased towards the production of Aβ1–40 at the detriment of Aβ1–42. Interestingly, detectable levels of both peptides occurred in cell homogenates, such detection is unlikely due to cross-reactivity with APP (according to the manufacturer). Therefore, we hypothesize two explanations for such intracellular content: either such measurement is indicative of some receptor-mediated uptake by BMECs as reported in the literature [15, 16, 42–45] or reflective of impaired vesicular trafficking (e.g., pinocytosis, caveolae) in PSEN1-BMECs. Therefore, our future investigation will focus on assessing the uptake and diffusion profile of Aβ peptides across such monolayers.

Interestingly, preliminary data suggest that these two pathological iPSC lines were also displaying a higher gene expression level for *BACE1* and *LRP1* genes (*data not shown*), which would indicate a possible alteration of the amyloidogenesis pathway and Aβ clearance at the brain endothelial cell as a whole. We are currently investigating a transcriptome analysis of these cells to identify putative gene networks associated with the observed PSEN1 and PSEN2 phenotypes.

These results also suggest that an exacerbated Aβ1–40 production and release in the vicinity of BMECs may impair their integrity. We speculate that Aβ1–40 may partially contribute to the phenotype observed in our iPSC lines. However, we cannot state that such impaired phenotype is solely due to higher Aβ levels alone. PSEN1- and PSEN2-BMECs showed very similar Aβ1–40 secretion levels (which would indicate similar γ-secretase activity), yet we also observed that PSEN1-mutant BMECs had a much worse phenotype than PSEN2-mutant which suggest other Aβ-independent signaling pathways may be affected. Therefore, our future directions will address the effect of Aβ peptides (both 1–40 and 1–42) on our iPSC-derived model of the BBB, as well as characterizing differences at the transcriptome levels between control, PSEN1, and PSEN2 BMECs.

In addition, γ-secretase has been documented as an important modulator of the canonical WNT signaling pathway [34, 46]. WNT signaling is an important pathway involved in the development and maintenance of the BBB [47, 48]. At this point, we cannot restrict and determine if the impairment of the BBB by PSEN1 is driven by an increase in Aβ production, or by an impairment of the endogenous WNT signaling.

In this study, we have reported that PSEN1-BMECs display a measurable impairment in their energetic profile, as such cells showed impaired glycolytic function, as well as mitochondrial function. Such impaired mitochondrial function was further supported by evidence of impaired mitochondrial membrane potential (as seen by JC-1 staining and flow cytometry data), which correlated with an increased oxidative stress status (as measured by intracellular ROS levels) in such cells compared to control-BMECs.

Preliminary data (unpublished results) by our group suggests that glucose homeostasis is essential for BMECs, as partial or complete glucose deprivation results in significant impairment of cell metabolism, glucose uptake, cell glycolytic capacity and ultimately an impaired barrier function within 24 h. Notably, such impaired energetic deficit appears not compensated by the addition of an alternative source of energy (e.g., ketone bodies, glutamine, etc.). Therefore, there is a need for further studies to better understand the bioenergetic and metabolic needs of the blood–brain barrier in regard to brain endothelial cells but also integrated with the neurovascular unit as a whole.

Furthermore, such issues seem not to be exclusive to impaired mitochondrial function, as impaired lysosomal acidification (as measured by acridine orange) was also observed in such cells. These two components playing essential roles in bioenergetic homeostasis as well as autophagy/mitophagy, which are documented cellular functions impaired in animal models harboring PSENs mutations [26, 28–30, 49]. These two features remain largely undocumented at the BBB despite their important contribution in neurological diseases and therefore raises the need for further investigation on the contribution of bioenergetics and autophagy in the brain endothelial cells physiology, and how such cellular functions can contribute to the dysfunction of the BBB during neurological diseases.

In this study, we primarily focused on the effect of mutated PSEN1 and PSEN2 genes on the barrier phenotype, with an approach centered on the brain endothelial cell. Such an approach comes with a caveat over the exclusion of cellular components of the neurovascular unit, which results in a limitation of our study. There is existing literature from our group [50], as well as from others [51], that astrocytes or neurons derived from diseased patients (Batten’s disease, Huntington’s disease) can have detrimental effects on barrier function when co-cultured with BMECs monolayers isolated from healthy patients.

Thus, a better understanding of how PSENs impact these pathways may increase interest in understanding the contribution of these pathways on the BBB dysfunction during neurological diseases.

## Conclusions

In conclusion, this study suggests that mutations in the *PSEN1* gene (rather than *PSEN2* gene) may be detrimental in the differentiation of iPSC-derived BMECs into functional monolayers, which is in the agreement with the existing literature. Such detrimental effects may be the consequence of the impairment of the brain endothelial cell integrity as a whole (decreased glucose uptake, impaired mitochondrial function, impaired autophagy….) rather than solely impaired barrier function.

Hence, our future goal is to strengthen this pilot study by the inclusion of additional iPSC lines from FAD patients, alongside the generation of isogenic control and PSEN1 mutant by gene editing) iPSC lines to better understand how the select number of genes associated with FAD impact the blood–brain barrier integrity. Such a study, along with our previous studies [50, 52], raises the importance of investigating the contribution of genetic disorders at the BBB, and the possible inclusion of a dysfunctional BBB in the pathophysiology of the disease.

## Supplementary Information

**Additional file 1: Fig. S1.** Phenotype of iPSC-derived neurons differentiated from the iPSC lines. Cells were differentiated into neurons following existing protocols [24, 53]. Neurons were stained against nestin (red), βIII-tubulin. DAPI was used for nuclear counterstaining. **Fig. S2. **Effect of PSEN1 and PSEN2 mutations on astrocytes glucose uptake and cell metabolic activity. (A) Glucose uptake assay in iPSC-derived astrocytes. Note the similar decrease in glucose uptake as observed in BMECs as well as the efficacy of GTI as a pharmacological inhibitor for GLUTs, as all three cell lines showed a significant decrease in glucose uptake. (B) Cell metabolic activity in astrocytes and neurons using an MTS-assay. Note the higher cell metabolic activity reported in PSEN1-neurons compared to other groups, such as higher metabolic rate being absent in astrocytes. (C) DCF levels in control BMECs following incubation with pyocyanin or N-acetyl-cysteine (NAC). **Fig. S3.** Effect of PSEN1 and PSEN2 mutations on iPSC-derived BMECs cell viability. Representative micrograph pictures of calcein-AM uptake in iPSC-derived BMECs from control, PSEN1, and PSEN2 iPSC-derived BMEC cells. Note the quasi-absence of ethidium homodimer-1 positive cells (red) as indicative of apoptotic cells. Bar scale = 200 µm.

## Data Availability

The data presented in this work is available upon request.
