# Supplementary material for: Presence of a mutation in PSEN1 or PSEN2 gene is associated with an impaired brain endothelial cell phenotype in vitro
Source: Fluids Barriers CNS. 2021 Jan 7;18:3. doi: 10.1186/s12987-020-00235-y (PMC7789219; doi:10.1186/s12987-020-00235-y)
Supplement: Supplementary file 1 — Additional file 1: Fig. S1. Phenotype of iPSC-derived neurons differentiated from the iPSC lines. Cells were differentiated into neurons following existing protocols [24, 53]. Neurons were stained against nestin (red), βIII-tubulin. DAPI was used for nuclear counterstaining. Fig. S2. Effect of PSEN1 and PSEN2 mutations on astrocytes glucose uptake and cell metabolic activity. (A) Glucose uptake assay in iPSC-derived astrocytes. Note the similar decrease in glucose uptake as observed in BMECs as well as the efficacy of GTI as a pharmacological inhibitor for GLUTs, as all three cell lines showed a significant decrease in glucose uptake. (B) Cell metabolic activity in astrocytes and neurons using an MTS-assay. Note the higher cell metabolic activity reported in PSEN1-neurons compared to other groups, such as higher metabolic rate being absent in astrocytes. (C) DCF levels in control BMECs following incubation with pyocyanin or N-acetyl-cysteine (NAC). Fig. S3. Effect of PSEN1 and PSEN2 mutations on iPSC-derived BMECs cell viability. Representative micrograph pictures of calcein-AM uptake in iPSC-derived BMECs from control, PSEN1, and PSEN2 iPSC-derived BMEC cells. Note the quasi-absence of ethidium homodimer-1 positive cells (red) as indicative of apoptotic cells. Bar scale = 200 µm. [file 12987_2020_235_MOESM1_ESM.pptx]

## Slide 1
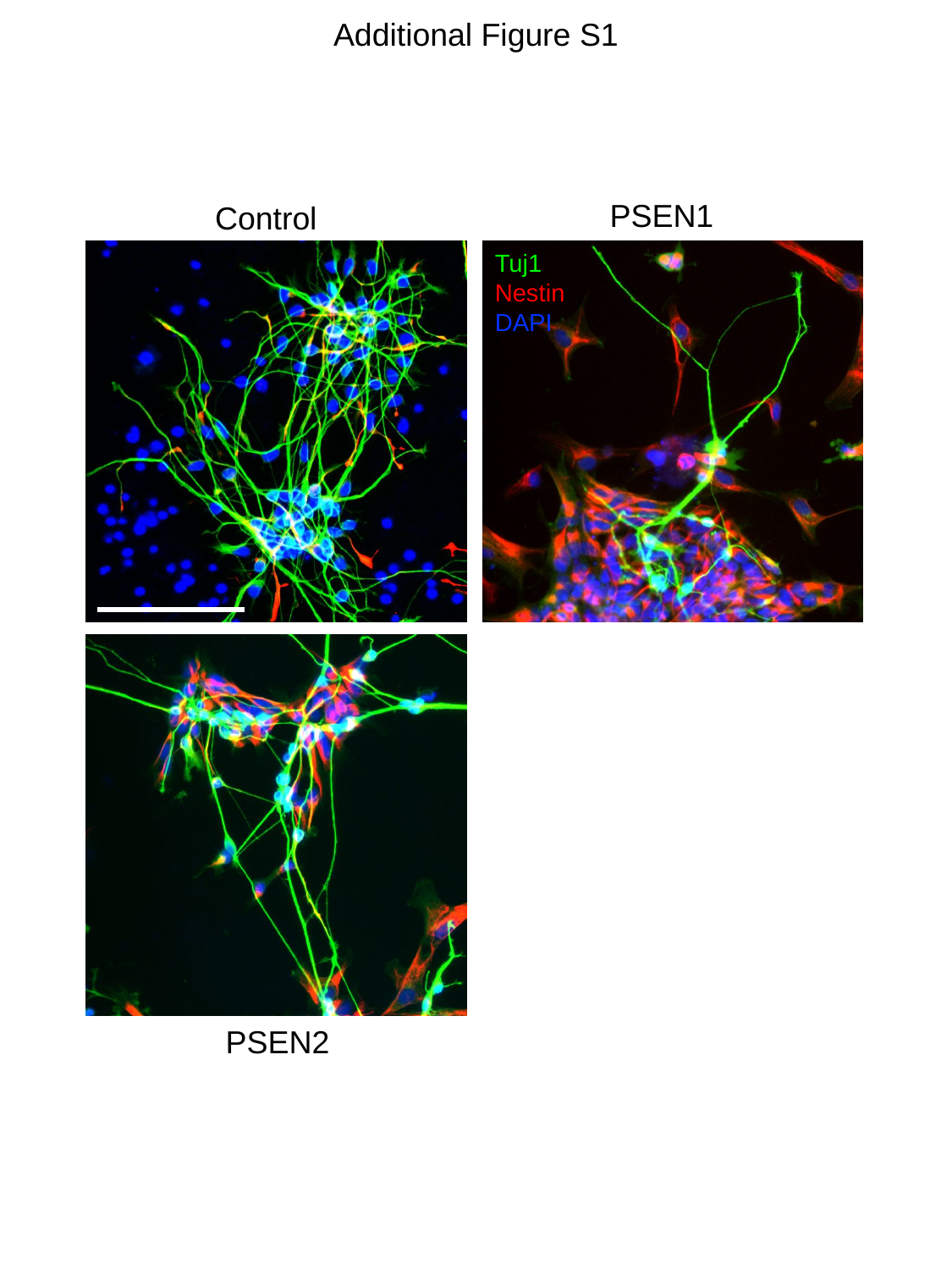

# Additional Figure S1
PSEN1
Control
Tuj1
Nestin
DAPI
PSEN2

## Slide 2
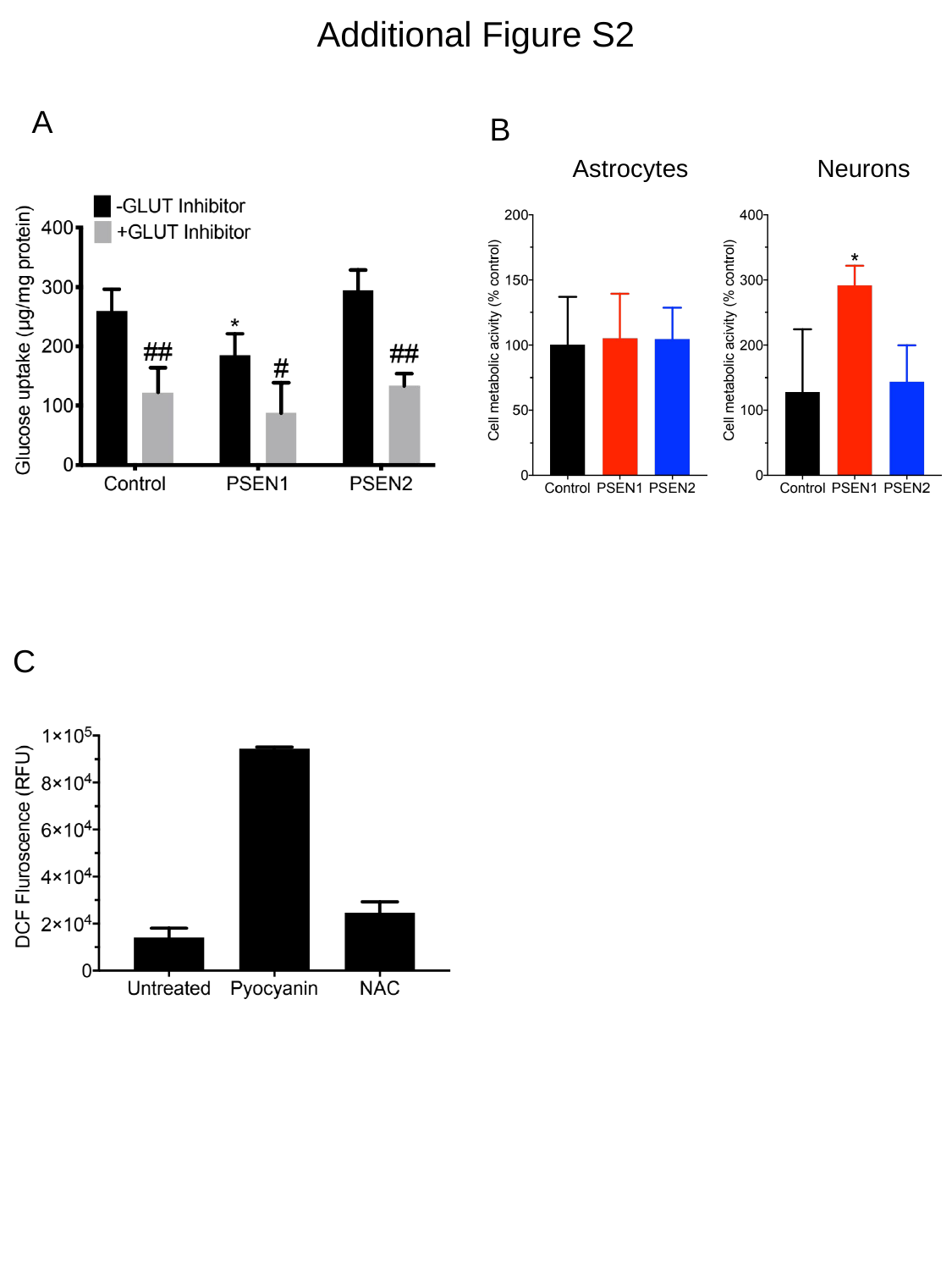

# Additional Figure S2
A
B
Astrocytes
Neurons
C

## Slide 3
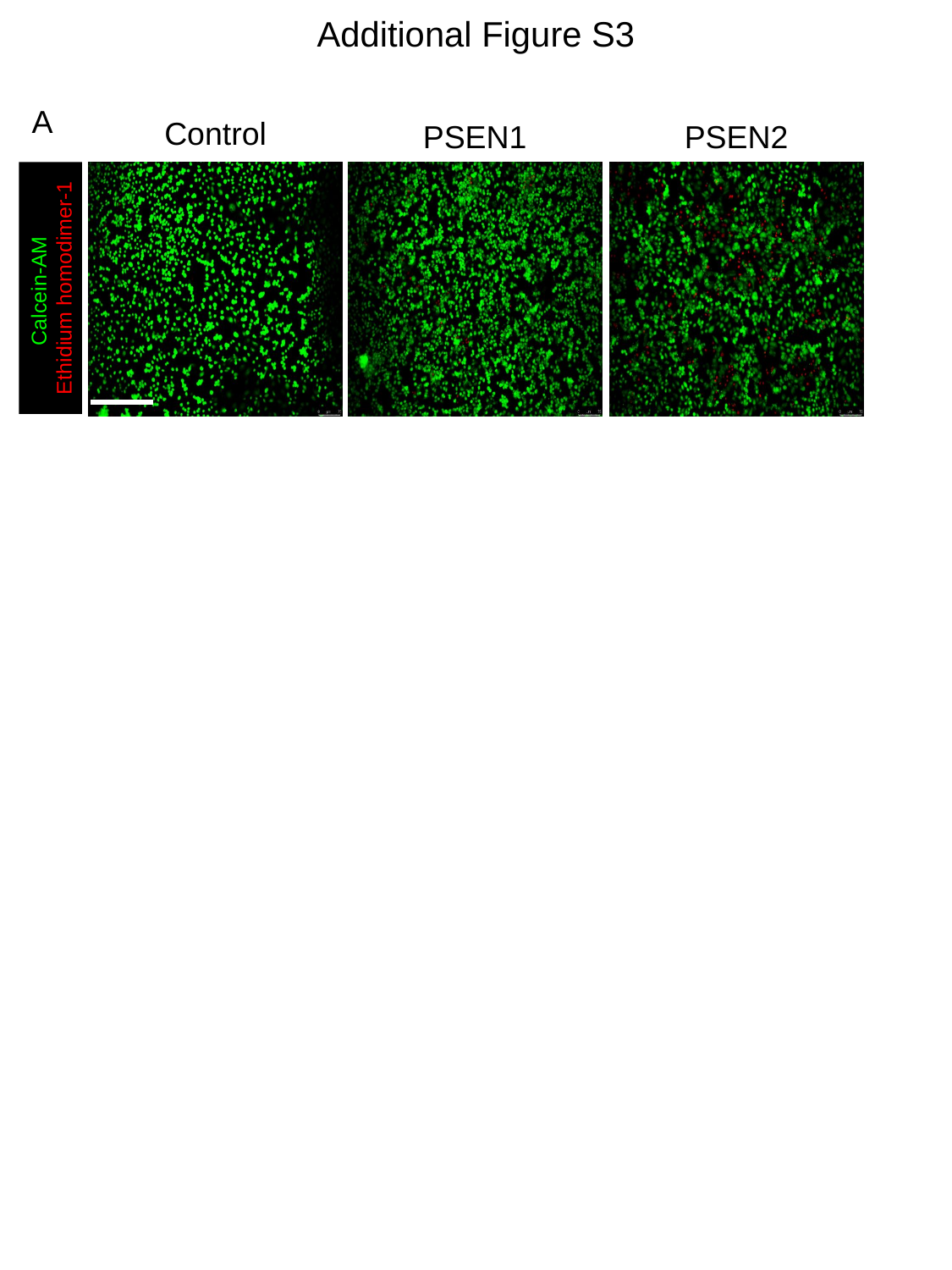

Additional Figure S3
A
Control
PSEN1
PSEN2
Calcein-AM
Ethidium homodimer-1
